# Neural Underpinnings of the Perception of Emotional States Derived From Biological Human Motion: A Review of Neuroimaging Research

**DOI:** 10.3389/fpsyg.2018.01763

**Published:** 2018-09-21

**Authors:** Julia Bachmann, Jörn Munzert, Britta Krüger

**Affiliations:** Neuromotor Behavior Laboratory, Department of Psychology and Sport Science, Justus-Liebig-University Giessen, Giessen, Germany

**Keywords:** emotion, emotion recognition, body movements, fMRI, point-light displays, AON, mentalizing

## Abstract

Research on the perception of biological human motion shows that people are able to infer emotional states by observing body movements. This article reviews the methodology applied in fMRI research on the neural representation of such emotion perception. Specifically, we ask how different stimulus qualities of bodily expressions, individual emotional valence, and task instructions may affect the neural representation of an emotional scene. The review demonstrates the involvement of a variety of brain areas, thereby indicating how well the human brain is adjusted to navigate in multiple social situations. All stimulus categories (i.e., full-light body displays, point-light displays, and avatars) can induce an emotional percept and are associated with increased activation in an extensive neural network. This network seems to be organized around areas belonging to the so-called action observation network (PMC, IFG, and IPL) and the mentalizing network (TPJ, TP, dmPFC, and lOFC) as well as areas processing body form and motion (e.g., EBA, FBA, and pSTS). Furthermore, emotion-processing brain sites such as the amygdala and the hypothalamus seem to play an important role during the observation of emotional body expressions. Whereas most brain regions clearly display an increased response to emotional body movements in general, some structures respond selectively to negative valence. Moreover, neural activation seems to depend on task characteristics, indicating that certain structures are activated even when attention is shifted away from emotional body movements.

## Introduction

As social beings, we spend the major part of our time interacting with others. Therefore, it is important for us to recognize and properly assess our conspecifics’ emotions so that we can respond to them appropriately. This is advantageous, because it enables us to approach people who seem to be in a good mood while avoiding contact with those who are angry, threatening, or dangerous.

Emotions can be transmitted via very different channels. Most research on emotion perception and processing has focused on emotions expressed via facial expression and/or prosody. However, these emotional channels are not the only source of input conveying emotionally relevant information. Another important human-emotion-expressing system that plays a central role in person recognition has come into the spotlight in recent years: the human body; that is, body language and body movements (de Gelder, 2006; de Gelder et al., 2014). Human body movements can convey emotions, and observers can infer the emotional state of individuals or interacting partners from such movements even when these are still at a distance and their faces are not clearly visible (Michalak et al., 2009, 2011; Lorey et al., 2012; Kaletsch et al., 2014b,a). Furthermore, because emotions are closely linked to actions (Frijda, 1986), the bodily expression of an emotion can act as a direct cue as to what might serve as an adequate behavioral response (de Gelder, 2006; de Gelder and Hortensius, 2014).

On a neural level, research has demonstrated that the perception of emotional bodily expressions is linked not only to the activation of areas important for emotional processing such as the amygdala (Hadjikhani and de Gelder, 2003), but also to action representations and motor responses (de Gelder et al., 2004; for reviews, see de Gelder, 2006; de Gelder et al., 2010). This underpins the important linkage between emotions and adequate (re-) actions (Frijda, 1986; Panksepp, 1998).

The paradigms applied to investigate the perception of emotional body movements and the underlying neural substrate differ widely. However, what all paradigms have in common is that they present emotional body movements while excluding facial information and other distracting variables. Nonetheless, the stimulus material applied in research varies substantially with respect to the available information on the observed moving body, thereby providing different advantages and supporting different research perspectives. For example, point-light displays (PLDs) provide only kinematic information stemming from just a few points representing the joints of the body (Johansson, 1973). This ensures that perception is not affected by confounding variables in the stimulus material such as attractiveness, sympathy, and cultural aspects (Hoffmann et al., 2010). An avatar, however, provides further information on the shape of a moving body as well as depth cues, thereby creating a more complex percept and approximating the natural richness of emotional interactions.

Against this background, the present review examines the common methodology in the research field addressing perception of emotional body movements. We have chosen to describe results from studies using moving stimuli (i.e., full-light displays, PLDs, and avatars) because our theoretical focus lies on the perceived and represented differences between the different categories of moving human body stimuli that possess emotional content. We believe that whether the observer views static whole-body images or bodies in movement makes a qualitative difference because, as, for example, Atkinson et al. (2004) have stated, it is especially the perceived intensity of body gestures that relies more on movement than on static form information. Against this background, our aim is to analyze research that offers insight into the neural representation of the perception of emotional states derived from kinematic and bodily cues of biological (human) motion. We discuss how emotional movement stimuli of different quality may modulate the underlying neural substrate of the observer’s perception. We further ask how depicted emotional valence (negative vs. positive) as well as different task instructions (implicit vs. explicit paradigms) might affect perception and its neural processing.

## Literature Search

**FIGURE 1:**
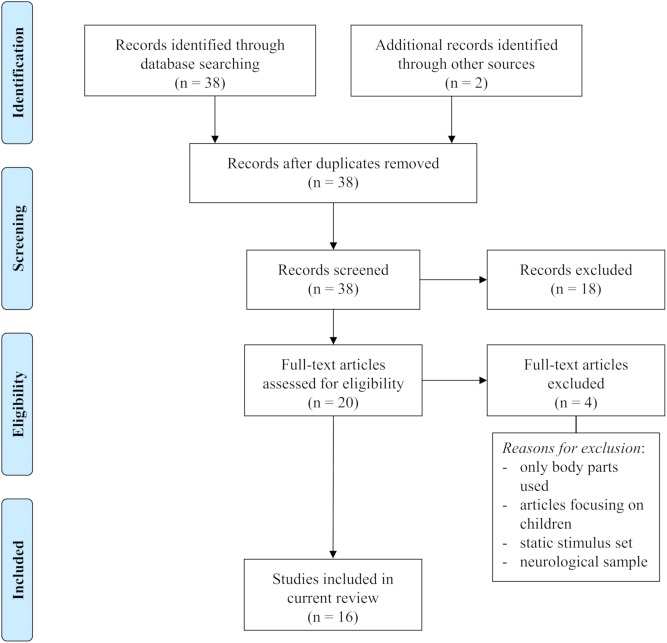
PRISMA flow diagram of literature selection. Adapted from Moher et al. (2009).

We identified and selected relevant studies (for selection process, see **Figure 1**) by searching in PubMed, Google Scholar, and Web of Science. We used several key terms in different variations, for instance fMRI, fNIRS, emotional brain, emotion, perception, point-light displays, body expressions, dynamic body, neural representation, body parts, biological motion, avatars, motion capture, emotional body language, kinematics, and emotion recognition. We then selected potential studies by examining their abstracts. Additionally, we scanned references retrieved from the selected literature for further articles on the topic. We included studies when (1) emotional body movements were used as stimulus material (i.e., full-light displays of whole bodies or body parts, point-light displays, avatars), (2) task requirements included emotion judgments or observation of emotional body movements, and (3) fMRI or fNIRS were used to assess brain activity. Based on these criteria, we selected a total of 16 studies for our review.

## The Neurophysiology of Perceiving Emotions from Emotional Body Movements

Neuroimaging studies on the perception of emotional body movements investigate the brain structures that play a key role not only in perception *per se* but also during the processes of emotion classification, valence judgments, and social perception in response to such movements.

Regarding the observation of human actions, the last two decades have seen the accumulation of a broad body of neuroimaging studies assessing the brain networks underlying the respective observation processes. One widely discussed underlying neural substrate of human action observation abilities is the so-called action observation network (AON). This is supposed to play a crucial role when observing the actions of others (for a meta-analysis, see Caspers et al., 2010). The AON embraces all brain areas activated by the mere observation of actions. According to a meta-analysis of 104 studies, enhanced activation during the observation of human actions is found in the inferior frontal gyrus (IFG), the dorsal and ventral premotor cortex (dPMC, vPMC), the supplementary motor area (SMA), the inferior parietal lobe (IPL), the superior parietal lobe (SPL), and the primary sensory cortex (SI) (Caspers et al., 2010). The AON is believed to integrate the observed actions of others with an individual’s personal motor repertoire. This suggests that the human brain understands actions by engaging in a motor simulation based on its own motor programs (Kilner et al., 2007). However, it has been asserted that understanding actions and their associated intentions cannot be purely motoric in nature. In addition, mental states such as a person’s feelings, emotions, and desires are attributed to other people in order to understand the intentions underlying their actions (Frith and Frith, 2003). In this regard, Frith and Frith (2006) have suggested that simulating motor or affective processes is only the first step toward inferring the other’s intentions and attitudes. Another process has been described as a mentalizing process or the employment of a “theory of mind” (ToM). This can be conceived as inferring another person’s mental state, and it is thought to be subserved primarily by four brain regions: the ventromedial prefrontal cortex (vmPFC), the dorsomedial prefrontal cortex (dmPFC), the precuneus, and the temporo-parietal junction (TPJ) (Centelles et al., 2011). Further structures such as the anterior superior temporal sulci (aSTS), the lateral orbitofrontal cortex (lOFC), and the amygdala may also play a major role (Gallagher and Frith, 2003; Saxe, 2006; Frith, 2007; Bedny et al., 2009; Carrington and Bailey, 2009). Turning to the processing of human body form, further regions are thought to be involved in processing human body movements. These include the extra striate body area (EBA) and the fusiform body area (FBA). Both have been reported to respond selectively to human bodies and body parts (Downing et al., 2001; Schwarzlose et al., 2005).

In the following sections, we present neuroimaging research based on the observation and valuation of emotional body movements. More precisely, we discuss three different stimulus types of emotional body movements—namely, full-light displays (videos), PLDs, and avatars. Hence, the main focus of the current review is on the underlying neural substrate of the processing of emotional body movements and the possible modulatory effects of different stimulus categories. Furthermore, we discuss the influence of stimulus valences (e.g., angry, fearful, happy) on brain sites that are mostly valence-independent as well as on sites that react preferentially to specific valences. In a last step, we discuss effects of different task instructions (e.g., implicit vs. explicit tasks) on neural activation.

### Stimulus Types

#### Full-Light Displays

Research on emotional body movements has frequently applied full-light displays of emotional body movements as stimuli. Although many studies have shown that emotional expressions can indeed be identified from the minimal kinematic information provided by, for instance, PLDs, Atkinson et al. (2004) have shown that the use of full-light displays leads to slightly better recognition accuracy rates for the specific emotional content. To construct stimuli, most studies asked non-professional or professional actors to enact different scenarios corresponding to emotional situations such as opening a door and reacting to someone or something that makes them angry (Pichon et al., 2008). Compared to more abstract displays of biological movements, this method clearly has greater ecological validity. However, it is harder to control and is therefore more susceptible to possible confounding factors such as the attractiveness of or sympathy toward the actor. Nonetheless, facial features and expressions are commonly removed and clothing is kept uniform (Grèzes et al., 2007) to ensure that these variables do not exert unwanted effects.

##### Neural correlates of observing full-light displays

**FIGURE 2:**
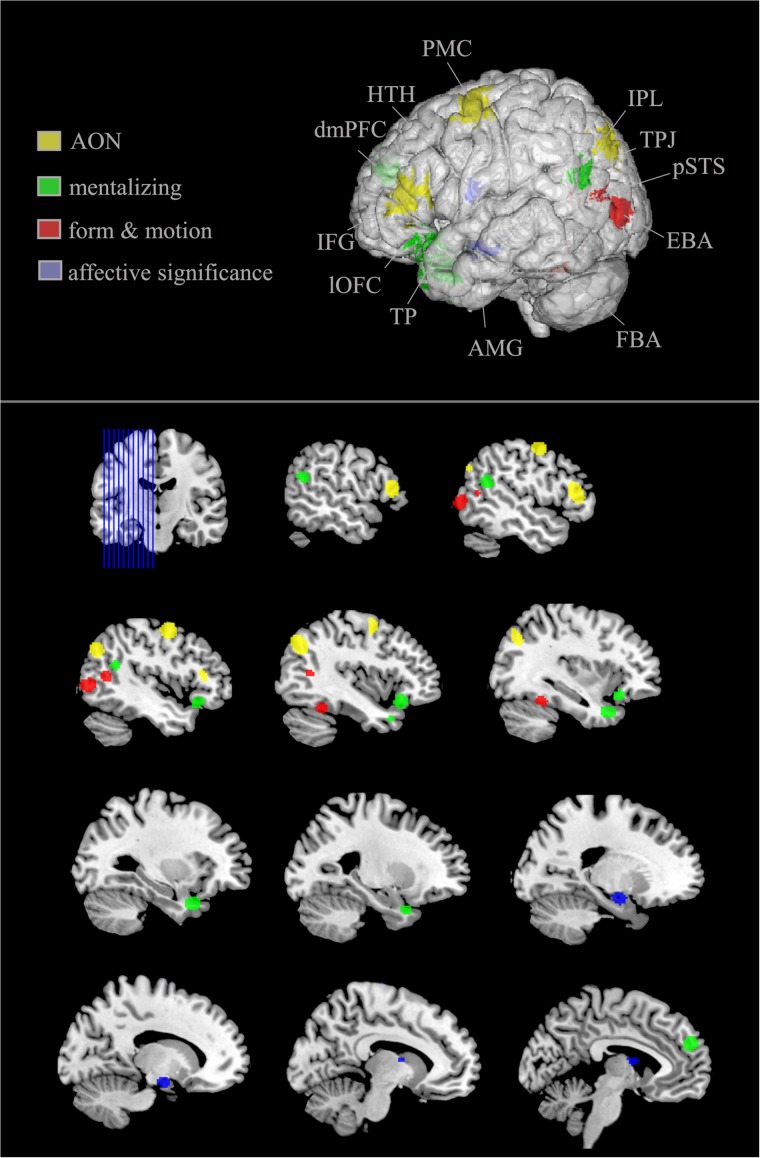
Brain structures preferentially recruited by emotional (vs. neutral) body movements. Generic figure; upper image shows left hemisphere view. Increased surface transparency for visibility of subcortical structures (i.e., amygdala, hypothalamus). AON (yellow), action observation network; includes PMC, premotor cortex; IFG, inferior frontal gyrus; IPL, inferior parietal lobule; mentalizing network (green) includes lOFC, lateral orbitofrontal cortex; dmPFC, dorsomedial prefrontal cortex; TPJ, temporoparietal junction; TP, temporal pole; form and motion associated areas (red) include pSTS, posterior superior temporal sulcus; EBA, extra striate body area; FBA, fusiform body area. Structures associated with affective significance and defensive reactions (purple) include AMG, amygdala; HTH, hypothalamus. Lower part of the image presents sagittal sections of the left hemisphere with each structure presented at least once.

Studies using full-light displays to investigate how emotional body movements are represented on a neural level have found an involvement of various structures across the brain (**Figure 2**). This network revolves around structures that code basal inputs such as form information and motor dynamics, but also ones on a higher cognitive level that evaluate the significance and meaning of actions as well as the intention behind the action.

##### Action observation network

Full-light displays of emotional movements activate several structures that are thought to be part of the AON. These include the inferior parietal lobule (IPL) and the inferior frontal gyrus (IFG) that both show increased activation for actions containing emotional expressions when contrasted directly with neutral actions (Sinke et al., 2010). Comparisons of the observation of emotional actions with the observation of neutral actions have also revealed increased activation within the premotor cortex (PMC), thereby indicating that this area also exhibits a particular sensitivity for emotional body movements (Grèzes et al., 2007; Pichon et al., 2008; Sinke et al., 2010). This involvement of AON-associated structures suggests that observing emotional movements might be mediated by a mental simulation process that draws on own motor representations of such movements in order to make sense of them. In this context, it is worth noting that a recent study by Jospe et al. (2018) has emphasized that this simulation process correlates with personality traits such as individual empathy levels. Their results showed that participants with high empathy scores utilize the embodiment or simulation process more optimally than participants with low empathy scores.

##### The role of the mentalizing network

As stated above, although the AON is thought to be at the core of understanding another person’s actions, the process of inferring the meaning behind actions has also been associated with so-called mentalizing processes (Frith and Frith, 2006). Within this framework, structures that commonly show an increased activation in response to emotional body movements compared to neutral actions include the temporo-parietal junction (TPJ) and the temporal pole (TP) (Pichon et al., 2008, 2009; Sinke et al., 2010). The TPJ is thought to be involved in reorienting processes, but has also been found to be active during mentalizing tasks and higher cognitive tasks such as perspective taking (Frith and Frith, 2006; Krall et al., 2015). It has been suggested that the temporal pole is a convergence zone in which knowledge from different modalities is integrated. For instance, knowledge about a person can be used to predict not only thoughts, feelings, and behavior that are most likely to occur within a given situation and vice versa, but also how moment-to-moment changes of a situation can alter these thoughts, feelings, and behavior (Frith and Frith, 2006). In this context, it is assumed that activation of the TP allows the observer to integrate the emotional behavior with the situational context in order to gain a meaningful understanding of it.

A more frontal region belonging to the mentalizing network, the medial prefrontal cortex (mPFC), has also repeatedly been found to be activated in persons observing and subsequently rating emotional body movements in terms of their emotional valence (e.g., neutral or angry) (Pichon et al., 2008, 2009, 2011; Sinke et al., 2010). The mPFC is considered to be associated with anticipating what another person is thinking or feeling. It has also been shown to be active when thinking about one’s own mental state. It is therefore postulated that the mPFC is used to interpret what a person is likely to be feeling in a given situation (Frith and Frith, 1999).

Finally, increased activation of the lateral orbitofrontal cortex (lOFC) is found in response to the observation of emotional (vs. neutral) body movements (Pichon et al., 2008, 2009, 2011; Sinke et al., 2010; Van den Stock et al., 2011). Activation of the lOFC has also been associated with mentalizing and particularly with ToM processes (Völlm et al., 2006) as well as with comparing non-matching social-emotional cues (i.e., social violations) with stored representations (Moll et al., 2005).

Hence, the mentalizing network seems to be responsive toward emotional content when judging dynamic movements. Considering the simultaneous involvement of AON-associated structures, the increased sensitivity of the mentalizing network to emotional actions supports the idea that they serve a common purpose—that is, to infer and interpret affective states from body movements.

##### Areas encoding motion and form information

One further structure that is believed to be critical in decoding human motion (Blake and Shiffrar, 2007) is the (posterior) superior temporal sulcus (pSTS). In addition to its activation being elicited by human motion *per se*, the pSTS has also often been shown to be activated by dynamic emotional expressions (Grèzes et al., 2007; Pichon et al., 2009, 2011; Kret et al., 2011; Van den Stock et al., 2011). Moreover, it is worth noting that the pSTS has frequently been discussed as part of the AON. Urgen et al. (2016), for instance, pointed out that during the visual processing of actions, the pSTS pools information from the visual cortex and passes that information to higher levels of the AON (e.g., the PMC) that code higher aspects of actions.

Aside from motion information, emotional body movements also provide form information. Previous research suggests that the extra striate body area (EBA), located in the posterior inferior temporal sulcus/middle temporal gyrus, is linked to body representation in general and responds selectively to emotional bodies compared to a variety of other objects (Downing et al., 2001; Peelen et al., 2007).

Moreover, empirical studies have demonstrated a higher sensitivity of the EBA for emotional compared to neutral body movements (Grèzes et al., 2007; Peelen et al., 2007; Pichon et al., 2009; Kret et al., 2011). In this context, it is worth noting that the fusiform body area (FBA), a structure of the ventral fusiform gyrus, is also especially sensitive for emotional body movements compared to neutral body movements (Pichon et al., 2009; Sinke et al., 2010; Kret et al., 2011).

Hence, brain areas associated with motion and form processing show increased neural responses especially to movements with emotional content. It has been proposed that this is an outcome of the modulation by another brain structure—namely, the amygdala (Pichon et al., 2009).

##### Affective significance and action preparation

When observing emotional body movements, it is important to be able to judge the respective affective significance of the observed movement in order to know how to respond appropriately. This process is believed to be conducted by the amygdala, a structure that is also tightly connected with structures that play a pivotal role in defense reactions and action preparation such as the hypothalamus and the PMC (Barbas et al., 2003). Seeking support for this idea, Sinke et al. (2010) investigated whether small differences in body movements (i.e., threatening vs. teasing body movements) would lead to differential brain activation. Their results showed that threatening stimuli elicited greater amygdala and hypothalamus activation than teasing stimuli. This suggests that when a stimulus is evaluated as potentially threatening, this information is passed on to structures that modulate vegetative preparedness such as the hypothalamus (Sinke et al., 2010).

##### An extensive network recruited by full-light displays

To conclude, studies using full-light displays show that emotion perception from body movements is associated with increased activation in a broad network of cortical and subcortical areas. Moreover, this activation involves numerous subprocesses. While being composed of basal processes such as recognizing human form and motion, it is also associated with structures such as the EBA, the FBA, and the pSTS along with higher cognitive processes such as assigning meaning to what has been observed (IPL, IFG, and PM). Moreover, a related process of making sense of the movement observed is to recognize the intention of the person carrying out the movement. These mentalizing processes are assumed to be carried out mainly by the TPJ, TP, mPFC, and lOFC. Finally, and not to be neglected, because it plays a pivotal role in processing emotional movements, evidence reveals amygdala and hypothalamus activation in response to displays of emotional body movements (*for an overview*, see **Figure 2**).

#### Point-Light Displays

Back in the early 1970s, Johansson (1973) described a technique for studying perception from biological motion that reduces stimulus information to a minimum. By attaching reflective patches to several anatomical landmarks and carefully controlling the light settings, high-intensity signals from the reflective patches were visible and could be recorded while subjects performed movements. Nowadays, methods are more advanced, and using motion capture software to create so-called point-light displays (PLDs) has become a popular method for assessing social and emotion perception from biological movements in both behavioral and fMRI paradigms (Atkinson et al., 2004, 2012; Heberlein and Saxe, 2005; Lorey et al., 2012). Behavioral studies have shown a variety of inferences that can be made based on the observation of PLDs. These include attractiveness, affect, and vulnerability (Gunns et al., 2002; Provost et al., 2008; Centelles et al., 2011). Although PLDs may have restricted ecological validity due to their level of abstraction, the method seems to be highly suitable for investigating social perception while eliminating potential confounds in this domain such as gender and attractiveness.

##### Neural correlates of observing point-light displays

The evidence base from point-light displays is considerably smaller than that from the broader body of studies using full-light displays. In the following, however, we discuss whether a reduction of visual information, leaving purely kinematic stimuli, might affect neural activation in the discussed regions of interest (ROIs).

##### The action observation network

Because research has been shown that the mere observation of PLDs without explicit emotional content recruits action observation networks (Saygin et al., 2004), it is reasonable to assume that emotional body movements, depicted as PLDs, will also evoke activity in AON regions. In support of this assumption, the observation of, among others, emotional PLD interactions (Centelles et al., 2011) revealed activation in the PMC, and IFG. Alaerts et al. (2013), for example, carried out fMRI scanning with typically developed as well autistic individuals while they were performing an emotion recognition task using PLDs and a control task in which they observed the same PLDs but were instructed to direct their attention toward color changes rather than emotional states. Their results demonstrated that, compared to fixation, emotion recognition from PLDs activated several fronto-parietal regions of the AON as well as the IPL. These data underpin the notion that even after a reduction of visual information, activation is still elicited in fronto-parietal regions of the AON subserving action and/or emotion processing as well as embodied cognition (Rizzolatti and Craighero, 2004).

##### The mentalizing network

When inferring affective states from PLDs, it seems that the mentalizing network also plays a key role, as previously reported for full-light displays. For example, the TPJ has been identified repeatedly as being activated by the observation of emotional PLDs (Heberlein and Saxe, 2005; Centelles et al., 2011). Heberlein and Saxe (2005), for instance, asked their participants to rate the fit between an emotion or trait word and a previously observed point-light video. They found that the TPJ showed increased activation regardless of whether participants inferred either affective states or personality traits. Furthermore, the dorsal part of the mPFC (dmPFC) and the lOFC have also been found to be activated during the observation of emotional PLD movements (Heberlein and Saxe, 2005; Centelles et al., 2011), implying that these regions may not be specifically sensitive to the reduction of visual information.

In contrast to the literature on full-light displays, the studies using PLDs reviewed here do not report an increased activation of the TP. However, this should be interpreted with caution, because the TP might not have been a predefined ROI in the reviewed studies. However, on the basis of the evidence discussed in the present review, we cannot rule out the possibility that the TP is more sensitive to other stimulus properties such as visual context and ecological information in full-light displays, and therefore not activated successfully by point-light displays of emotional kinematics.

##### Motion and form information

Although PLDs do not include the same information on shape such as soft tissue form and motion, they show a similar pattern of neural activation within, for instance, areas associated with human body shape and motion. For example, Atkinson et al. (2012) compared neural responses to emotional (i.e., angry, happy) and non-emotional actions depicted as PLDs. They found that all human–motion- and form-information-associated areas (i.e., EBA; FBA, and pSTS) responded preferentially to emotional compared to non-emotional PLDs. Within this framework, it is worth noting that a study investigating neural responses to various complex motion stimuli showed that the STS reacted slightly more strongly to videos of humans performing different whole-body motions than to point-light displays of such motions (Beauchamp et al., 2003). However, a direct comparison between emotional full-light displays and PLD movements has yet to be conducted.

##### Affective significance and action preparation

Interestingly, no activation has been reported in either the amygdala or the hypothalamus when comparing emotional PLDs with neutral stimuli (Heberlein and Saxe, 2005; Centelles et al., 2011; Atkinson et al., 2012). Both groups of nuclei have been implicated in motor vigilance to support reflexive defensive behaviors, typically in response to threatening stimuli portraying, for example, anger (Pichon et al., 2011). Although the stimuli used in the present studies included negative emotional displays and the emotion could be categorized correctly, the lack of activation in these areas may be due to the level of abstractness of the stimuli. Another possibility is that predefined regions were used for the fMRI analyses and further activated regions may have been missed.

##### Similar activation pattern elicited by PLDs

Although PLDs contain only a minimum of kinematic information, studies using PLDs confirm that emotional point-light body expressions also modulate neural activation successfully in most of the areas that have previously been discussed as being relevant for the processing of emotional movements on the basis of findings using full-light body stimuli. These areas revolve around structures that are often associated with motion and form processing (pSTS, EBA, and FBA), the AON (IPL, IFG, and PMC), and mentalizing processes (dmPFC, lOFC, and TPJ). No activation has been reported in the amygdala and the hypothalamus, indicating that PLDs may not be suited to elicit defensive responses in the observer. However, it should be emphasized that most studies analyzed predefined ROIs and this limits their comparability.

#### Avatars

Recent years have seen increased interest in a third class of emotional movement stimuli. Whereas PLDs have been criticized because their level of abstractness may affect their ecological validity, full-light displays are restricted in their controllability with respect to confounding variables such as gender or age (Goldberg et al., 2015). Therefore, researchers developed an intermediate stimulus category: dynamic avatars. de Borst and de Gelder (2015) have argued that avatars may evoke differential processing (e.g., better discrimination), and this, in turn, may affect underlying brain activity. Because this is a relatively new paradigm, the body of neurophysiological research on emotion perception from avatars is still quite small. However, there are two studies that have investigated emotion perception by using avatars within a neurophysiological framework.

##### Neural correlates of observing avatars

Because only two studies have used paradigms including this type of stimulus, we decided to summarize their findings without subdividing this section according to different networks or brain regions.

Both studies used avatars displaying different emotional gait patterns (i.e., happy, angry, fearful, sad, or neutral). Participants were then asked to classify the presented emotional state during fMRI scanning (Schneider et al., 2014; Goldberg et al., 2015). Results showed that, in addition to regions previously associated with form and motion processing (i.e., EBA, FBA, and pSTS), there was activation within the fusiform face area (FFA) (Goldberg et al., 2015). The FFA is an area that responds selectively to faces (Atkinson et al., 2012). This result is quite surprising, in view of the fact that all avatars were “faceless.” However, it could suggest that face completion effects may occur even when detailed facial information is missing (Goldberg et al., 2015).

Moreover, emotional gait patterns also elicited activation in two structures of the AON: the IPL and the IFG (Goldberg et al., 2015). However, no activation in the PMC was reported.

Regarding the mentalizing network, activation was observed in the TPJ and lOFC, yet no activation was reported in the dmPFC. Furthermore, emotional (vs. neutral) gait elicited an increase in activation in the amygdala but not in the hypothalamus (Goldberg et al., 2015).

Thus, observation of emotional gait patterns of avatars also elicited increased neural activation in the networks discussed within the framework of the processing of emotional body movements. However, it has to be considered that participants observed emotional gait patterns displayed by avatars instead of complex interactions or emotional gestures, as described previously. Therefore, the studies cannot be compared directly because they used different stimuli. However, the lack of activation in the PMC and hypothalamus support the notion that, perhaps, avatars displaying emotional gait are not able to elicit defensive behaviors in the observer, perhaps to the material’s level of abstractness.

### The Influence of Emotional Valence

The following paragraph describes possible modulatory effects of different emotional valences on the previously identified structures that appear to be involved in processing affect from body movements. Emotional valence means the intrinsic attractiveness (positive valence) or averseness (negative valence) of an event, object, or situation (Frijda, 1986). The term can also be used to characterize and categorize specific emotions. For example, anger and fear are considered to have *negative valence*, whereas joy, for example, has *positive valence*.

Moreover, it has been discussed whether some structures may even represent activation in favor of individual emotions (e.g., anger and fear) that can, for instance, be assigned to the same valence category (e.g., negative).

#### Valence effects

Indeed, several brain structures have been identified as responding more strongly to certain valences. One of these structures is the EBA. Schneider et al. (2014) reported that the EBA was not significantly more activated during the display of avatars showing a happy gait pattern compared to neutral gait. In contrast, an increase in activation was reported for angry, sad, and fearful stimuli (i.e., emotions with a negative valence) compared to neutral ones. However, Peelen et al. (2007) did not report such exclusiveness for negative valence in the EBA, because they found an increased activation elicited by not only angry, fearful, disgusted body movements but also happy ones during the observation of full-light videos. This is in line with Atkinson et al. (2012) findings reporting enhanced activation in the EBA for angry as well as happy (vs. neutral) body movements. Schneider et al. (2014) argued, however, that happy walks were the most difficult for their participants to identify. Moreover, there was a slightly positive correlation between recognition accuracy and activation in the EBA, suggesting that better emotion recognition was also associated with increased neural activation. Therefore, they concluded that happy avatar walks may have lacked emotional expressiveness and therefore failed to induce a significant increase in cortical activation (Schneider et al., 2014).

Regarding AON-associated structures, preferential activation for negative valence was found within the PMC as well as within the IPL. This effect was revealed by a direct comparison of threatening (i.e., negative) vs. teasing (i.e., positive) stimuli (Sinke et al., 2010).

A similar preference for stimuli with a negative valence (anger and fear vs. neutral stimuli) was found within the TPJ, a region associated with mentalizing processes (Centelles et al., 2011). In support of these findings, Schneider et al. (2014) also found a selectivity of the TPJ for negative stimuli—that is, avatars displaying an angry, sad, or fearful gait pattern. This is supported by a direct contrast between threatening and teasing stimuli in which threatening stimuli elicited greater activation (Pichon et al., 2008, 2009).

### Differential Activation Between Emotions of One Valence Category?

Beyond a preferential activation for different valences (i.e., positive vs. negative), several studies have examined whether emotions of the same valence categories elicit differential neural activations. Against this background, the EBA and FBA present such a preference for certain emotions. For example, Peelen et al. (2007) showed that the EBA was modulated most effectively by anger, disgust, and, to a lesser degree, fear. The EBA, however, did not show increased activation in response to body movements portraying sadness. The FBA showed a similar pattern. However, fear and sadness both failed to elicit an increased response (Peelen et al., 2007). In support of this, Pichon et al. (2009) identified the FBA as being preferentially activated by anger compared to fear or neutral body movements.

Peelen et al. (2010) applied a multivariate approach to investigate whether neural patterns in the STS display emotion specificity. They found that neural pattern within the STS discriminated between different emotion categories, allowing for a distinction between emotions such as anger, disgust, fear, and sadness based on their neural patterns. This relatively new method of fMRI data analysis reveals that not only overall activity changes in brain regions but rather a more distributed response pattern within one brain region indicate the response to observed stimuli (Haxby et al., 2001; Haynes and Rees, 2005; Kriegeskorte et al., 2008).

Regarding areas belonging to the AON, evidence is quite sparse. Comparing anger and fear directly, the PMC, however, also reveals a preferential activation for angry body motion (Pichon et al., 2009).

Mentalizing-associated structures such as the TPJ show a preferential activation for angry compared to fearful body motion that becomes visible in the supramarginal TPJ. However, a reverse pattern has been identified in the superior temporal part of the TPJ, in which a greater response to fearful (vs. angry) stimuli was elicited (Pichon et al., 2009). For the temporal pole, evidence is also largely homogeneous.

Both studies that investigated emotion selectivity reported a preferential activation in response to angry body movements compared to fearful or neutral ones (Pichon et al., 2008, 2009).

The same holds true for frontal areas—that is, the dmPFC and lOFC. Both structures have been identified as displaying a greater BOLD response during the observation of angry compared to fearful stimuli (Pichon et al., 2009). This finding is in line with the results of multivariate analyses by Peelen et al. (2010) in which the individual emotions (i.e., anger, disgust, fear, and sadness) could be distinguished based on their activation patterns, thereby indicating an “emotion specificity” in the mPFC.

Finally, the amygdala showed increased activation during the observation of angry as well as happy body movements, whereas movements displaying disgust, fear, and sadness (all compared with neutral stimuli) had no effect (Peelen et al., 2007). The authors suggested that this effect might be attributable to low levels of arousal induced by disgust, fear, and sadness compared to happiness and anger. Arousal dimensions have been highlighted in the context of amygdala activation, indicating that higher intensities correlate with higher activation magnitudes (Anderson et al., 2003).

In sum, there is a clear trend toward preferential activation in response to negative stimuli and, presumably, stimuli that induce high levels of arousal. Such emotional modulation has been postulated to originate in subcortical structures such as the amygdala (Amaral et al., 2003). However, a combined continuous theta burst stimulation (cTBS)-fMRI study by Engelen et al. (2018) found that when regions belonging to the AON (i.e., PMC and IPL) were stimulated, activation of the amygdala increased during the observation of threatening compared to neutral stimuli. Without stimulation, amygdala activation did not differ significantly between the two conditions. The authors suggested that this effect could be attributable to functional connectivity processes between the amygdala and structures of the AON (Engelen et al., 2018). However, the exact circuits and feedback processes have yet to be established. From an evolutionary perspective, an argument in favor of a preferential activation caused by threatening stimuli seems plausible, because identifying negative emotions might be more relevant for the organism so that it can quickly initiate a defense response. However, it should be emphasized that multivariate studies are more likely to shed light on whether certain structures produce emotion-specific activations. Whereas emotions of the same valence category may not elicit significantly different activation magnitudes, they may well be represented within a different pattern of activation (Peelen et al., 2010).

### Task Specificity

#### Experimental Paradigms

A variety of task paradigms using different types of stimuli and experimental instructions have been used to uncover the neural basis of emotion perception. For instance, within explicit observation paradigms, participants have been instructed to observe a short video clip displaying emotional body movements. In the next step, they are commonly asked to rate the video sequence they have seen in terms of its valence (emotional vs. neutral), or emotional intensity (e.g., “angry? 1: a little, 2: quite, 3: very much”), or make no judgment to ensure that the trials of interest are not confounded by motor responses. In the latter case, participants are instructed to press a button during control stimuli trials (e.g., upside down, oddball) to control for their attention.

Another popular method includes combined explicit and implicit observation paradigms. Within experimental runs, stimuli are blocked by task and alternate between series of explicit and implicit recognition. During explicit conditions, participants are asked to rate the emotion (e.g., fear/anger/neutral) displayed in the video sequence. During implicit conditions, they observe the same video sequences, but pay attention to colored dots or video frames that appeared during the videos. Following the sequence, participants are instructed to make a judgment about the color (Pichon et al., 2009; Alaerts et al., 2013).

Often, the implicit task condition serves merely as a control task. However, it does allow an investigation of the brain areas that may or may not be equally involved in conscious as well as non-conscious emotion processing.

#### Task Effects on Neural Activity

The extensive neural network involved in the perception of emotional body movements seems to be largely insensitive to a reduction of the visual properties of the observed moving stimulus. Nonetheless, modulatory effects regarding varying task requirements (implicit vs. explicit paradigm) can be observed.

Structures that appear to be preferentially activated during explicit emotion recognition include the pSTS and FBA (Pichon et al., 2011; Atkinson et al., 2012; Alaerts et al., 2013). For example, Alaerts et al. (2013) found that several regions, including bilateral pSTS, were activated more strongly during an explicit emotion recognition task compared to the implicit processing of emotional content during the control task. Further support stems from Atkinson et al. (2012) who directly compared an explicit emotion judgment task with a color naming control task. They also identified the pSTS as being more active during the explicit task. Thus, the STS seems to play a substantial role in the conscious perception of biological movement and dynamic emotional expressions (Pichon et al., 2008; Van den Stock et al., 2011).

Similarly, brain areas associated with mentalizing and ToM processes, such as the dmPFC, lOFC, and TPJ, responded preferentially during explicit emotion recognition (Sinke et al., 2010; Pichon et al., 2011). This may highlight that mentalizing processes are non-automatic and therefore require conscious attention.

When considering AON-related areas, the IFG also responded preferentially within explicit recognition trials across all study designs (Sinke et al., 2010; Pichon et al., 2011), whereas the PMC seemed to be equally responsive during both explicit and implicit trials. In contrast to brain structures in which activity seems to be modulated by explicit–implicit task demands, the PMC appears to be independent from attention control. This notion is underpinned in a study conducted by Van den Stock et al. (2011). They reported activity in the PMC also during conscious and non-conscious perception of emotions expressed via body movements in a patient with cortical blindsight.

Pichon et al. (2011) reasoned that especially the PMC may be involved in sustaining motor vigilance and also supporting reflexive defensive behaviors. The authors interpreted this finding as support for the view that emotions are essentially action tendencies that help the organism to adapt to environmental demands. Within this framework, recent evidence suggests that these activations more probably reflect automatic preparation for action (Pichon et al., 2008).

Two further structures are postulated to be involved in core processes of emotion recognition such as decoding affective significance and action preparation: the amygdala and the hypothalamus. Both have been shown to respond equally during attention and inattention (Pichon et al., 2011). In this regard, an interesting study on a patient suffering from cortical blindness (Van den Stock et al., 2011) investigated the neural correlates of residual visual perception for dynamic whole-body emotional actions that were presented in this patient’s intact and blind visual hemifield. Activations specific for blind hemifield presentation of angry compared with neutral actions were found in subcortical areas (e.g., the amygdala), thereby showing a selective involvement of these subcortical structures in non-conscious visual emotion perception. This is underpinned by a recent review from Diano et al. (2017) suggesting that evidence tends to support an amygdala response during inattention. However, they also emphasized that the results depend strongly on the choice of paradigm. In fact, Van den Stock et al. (2011) also reasoned that the absence of significant activity in the amygdala during conscious perception of emotional movements does not necessarily mean that the amygdala is not involved in conscious processing, because standard fMRI methods might not be able to make a fine-grained distinction of the possibly different temporal profiles of activation occurring in various brain areas.

To summarize, the reviewed data show that the different cortical and subcortical areas involved in the processing of emotional body movements show a selective involvement in the explicit or implicit processing of emotional body movements.

## Conclusion

The present review demonstrates that a variety of brain areas are involved in the perception and processing of emotional bodily expressions, indicating that the human brain is well adjusted to navigate in multiple emotional situations.

In general, it can be stated that all stimulus categories may induce an emotional percept and are associated with increased activation in a broad network of motor regions and/or in regions associated with emotional processing. It can be stated further that the emotional content of the moving stimuli matters. Thus, an emotional stimulus seems to elicit an enhanced neural response compared to a rather neutral one (Grèzes et al., 2007; Peelen et al., 2007; Pichon et al., 2008). The respective neural network seems to be organized around areas belonging to the so-called AON (PMC, IFG, and IPL), to the mentalizing network (TPJ, TP, dmPFC, and lOFC), and to areas processing body form and motion such as the EBA, FBA, and pSTS. Furthermore, emotion-processing brain sites such as the AMG and the hypothalamus seem to play an important role during the observation of emotional body expressions. For an overview of brain regions that are preferentially recruited during emotional (vs. neutral) body movements across all stimulus categories see **Supplementary Table 1**.

Regarding the different stimulus qualities, it can be concluded that both the perception and the associated neural activation pattern are partly modulated by the stimulus category in the following ways: (1) The observation of the most naturalistic full-light displays is linked to increased activation in the most extensive network including areas belonging to the AON (PMC, IFG, and IPL), the mentalizing network (TPJ, TP, dmPFC, and lOFC), body form and motion processing areas (EBA, FBA, and pSTS), and emotion-processing areas (amygdala and hypothalamus). (2) The observation of PLDs is associated specifically with increased activation in areas associated with processing motor (IPL, IFG, and PMC) and body information (EBA, FBA, and pSTS) as well as inferring mental states and intentions (dmPFC, lOFC, and TPJ). Increased amygdala activation, for example, was found only during the observation of full-light displays and avatars, indicating that the impression of animacy and body shape seems to have an increased impact on areas associated with emotional processing. Moreover, studies using PLDs and avatars did not report hypothalamus activation. This may suggest that, perhaps due to the level of abstractness or lack of realism, these stimulus categories do not elicit a preparation for possible defensive behaviors. However, this finding has to be viewed in light of the fact that all these PLD and avatar studies also used predefined ROIs for their analyses.

Regarding emotional valence, it is especially negative emotional content (such as anger or threat) that seems to be associated with increased activation of structures belonging to several networks (i.e., the STS, PMC, TPJ, dmPFC, and lOFC). This suggests that an adequate recognition as well as an adequate response to those who are angry, threatening, or dangerous may be of vital significance for self-protection and is therefore processed preferentially. Another possibility introduced by Peelen et al. (2010) is the idea that different emotions present different neural patterns, because the identified regions contain neurons that selectively represent emotion categories on an abstract level. Finally, attention matters. Whereas the PMC, the hypothalamus, and the amygdala have been shown to be equally responsive during explicit and implicit tasks, other structures, especially those associated with the mentalizing network (i.e., TPJ, dmPFC, and lOFC), respond only when participants have to judge emotional content explicitly.

However, it has to be noted that future studies need to investigate the different stimulus categories systematically within one design, because the results presented here stem from studies using a variety of designs, tasks, and statistics, and this indubitably contributes to their diversity. A further issue is that most studies used *a priori* defined search volumes. Therefore, it remains unclear whether further brain sites might process the observation and recognition of emotional body movements. Finally, the studies presented here used mostly traditional forms of fMRI data analysis that indicate only overall activity changes in brain regions in response to the observed stimuli. Newer approaches such as multivariate decoding (Haxby et al., 2001; Haynes and Rees, 2005; Kriegeskorte et al., 2008) might allow the investigation of the representational content of neuronal population codes and the identification of more distributed response patterns linked to a given stimulus. An increased usage of these methods will certainly further our understanding.

## Author Contributions

JB, JM, and BK contributed to the conception and design of the review. JB created and organized the database. All authors wrote the first draft of the manuscript, wrote sections and contributed to manuscript revision, read, and approved the submitted version.

## Conflict of Interest Statement

The authors declare that the research was conducted in the absence of any commercial or financial relationships that could be construed as a potential conflict of interest.
