# Supplementary material for: Neural Underpinnings of the Perception of Emotional States Derived From Biological Human Motion: A Review of Neuroimaging Research
Source: Front Psychol. 2018 Sep 21;9:1763. doi: 10.3389/fpsyg.2018.01763 (PMC6160569; doi:10.3389/fpsyg.2018.01763)
Supplement: Supplementary file 1 [file Data_Sheet_1.PDF]

Table 1

Brain regions preferentially recruited during the recognition of emotional (vs. neutral) stimuli (full-light displays, point-light displays, avatars)

		Brain regions												
Author /Year	***Emo-tion***	***AMG***	***PMC***	***(p)STS***	***EBA***	***FFA***	***FBA /FG***	***TPJ***	***TP***	***HTH***	***lOFC***	***(d)m PFC***	***IFG***	***IPL***
**Full-light displays**														
Peelen et al. (2007)	angry, disgust ed, happy	x			x	x	x							
Grèzes et al. (2007)	fearful		x	x	x			x						
Pichon et al. (2008)	angry		x	x	x		x	x	x	x	x	x		
Pichon et al. (2009)	fearful, angry	x		x	x		x	x	x		x	x	x	
Sinke et al. (2010)	threat, tease	x	x	x	x		x	x	x	x	x	x	x	x

Peelen et al. (2010)	disgusted, fearful, happy, sad			x								x		
Kret et al. (2011)	threatened			x	x		x	x						
Van den Stock et al. (2011)	angry	x		x			x				x		x	
Pichon et al. (2011)	angry, fearful	x	x	x	x		x	x		x	x	x	x	
Engelen et al. (2018)	angry	x					x							
**Point-light displays**														
Heberlein & Saxe (2005)	happy, sad angry, afraid							x				x		
Centelles et al. (2010)	(emo-tional) social inter-action		x	x			x	x			x	x	x	

Atkinson et al. (2012)	angry, happy			x	x	x								
Alaerts et al. (2014)	angry, happy, sad			x										x
**Avatars**														
Schneider et al., (2014)	fear, happiness, sadness, anger			x	x	x		x						
Goldberg et al., (2015)	fear, happi-ness, sadness, anger	x			x		x				x		x	x

Notes: Inclusion criteria were the identification of activated regions by more than one study; regions had to be preferentially activated by emotional stimuli (as compared to neutral stimuli); explicit and implicit conditions were included given that emotional stimuli were still visible (although attention may have been shifted to another task (i.e. Sinke et al., 2010)); analyses included dynamic stimuli (as compared to static stimuli). AMG: amygdala; PMC: premotor cortices; (p)STS: (posterior) superior temporal sulcus; EBA: extra striate body area; FFA: fusiform face area; FBA/FF: fusiform body area/fusiform gyrus; TPJ: temporo-parietal junction; TP: temporal pole; HTH: hypothalamus; lOFC: orbitofrontal cortex (incl. orbitofrontal gyrus); (d)mPFC: (dorso) medial prefrontal cortex; IFG: inferior frontal gyrus.
